# A Systematic Review of Early versus Late Closure of Loop Ileostomy

**DOI:** 10.1155/2020/9876527

**Published:** 2020-08-31

**Authors:** Ahmed A. Aljorfi, Abdulhameed H. Alkhamis

**Affiliations:** ^1^Department of Surgery, Western Health and Social Care Trust, BT74 6DN, Londonderry, UK; ^2^Department of Family Medicine, Doha Primary Health Care Centre, Ministry of Health, Dhahran 34454, Saudi Arabia

## Abstract

**Introduction:**

A Loop ileostomy is one of the most common techniques used in colorectal surgery to establish a reversible faecal diversion and bypass the large bowels, in order to protect either a downstream colorectal anastomosis or a coloanal anastomosis. However, it is a procedure that can cause a plethora of complications including long term ones such as the psychological effects. Currently, there is no consensus regarding the optimal time to perform closure of a loop ileostomy. Some studies suggested the early reversal of ileostomy procedure as a solution to reduce these complications. This study aims to review the available literature in order to ascertain the benefits behind early closure of loop ileostomy.

**Methods:**

The literature was searched for all studies that included a comparison between the outcomes of early and late closure of loop ileostomy in terms of morbidity, mortality, or quality of life, where available. Early closure of loop ileostomy is defined as closure less than three months and late as more than three months, in accordance with conventional literature. The resultant articles were filtered using our inclusion and exclusion criteria. Finally, the remaining articles were assessed for quality and their results were compared to one another in order to draw our conclusions. *Results and Discussion*. The results were slightly inclined toward early closure of loop ileostomy. However, there were limitations of the studies reviewed, including the heterogenicity of studies, selection bias, lack of clear definition of measured outcomes, and small sample size. Taking that into consideration, the results of early closure of loop ileostomies in the selected patients were promising and require further investigation.

## 1. Introduction

A loop ileostomy is one of the most common techniques used in colorectal surgery to establish a reversible faecal diversion and bypass the large bowels, in order to protect either a downstream colorectal anastomosis or a coloanal anastomosis [1, 2]. However, in spite of its potential benefits, it is worth noting that the incidence of complications, in general, is very variable and some studies estimated it between 14% and 79% [3]. Complications that arise from stoma formation can be divided into early and late. To better understand how these complications may arise, it is essential to discuss the steps involved in the procedures of a loop ileostomy's formation.

Loop ileostomy procedure is as follows [2]:

- The procedure starts by mobilizing a well-vascularized segment of the small bowel.
- This is followed by a creation of a circular incision of 2 to 4 cm in a predetermined site.
- While protecting the rest of the abdominal contents, the skin disc is resected and monopolar diathermy is used to deepen the incision to the anterior rectus sheath with the help of blunt retractors.
- At that level, the fascia is divided and the muscle is separated using blunt dissection.
- This will lead to the posterior sheath, which should be divided using diathermy until we are through the peritoneum.
- These steps have to be done while keeping in mind that the size of the internal incision should be enough to deliver the bowel without resulting in a parastomal hernia thereafter.
- The next step involves passing a finger through the defect to preserve the tract and to guide a babcock to clamp the bowel segment of interest.
- This is followed by pushing the clamped segment gently through the created defect via the guidance of the babcock clamp.
- Once this step is done, the main laparotomy incision can be closed and covered to minimize contamination of the wound.
- The next stage of the procedure involves maturing the stoma, in other words creating a spout by everting the edges of the stoma. This technique serves to protect the skin surrounding the stoma and to prevent distal strictures formation.
- It is worth noting that, in the case of loop ileostomies, a bridge is placed under the posterior wall of the stoma to prevent it from retracting [2].

Early complications, by definition, are those occurring within three months of the stoma creation. These include abscess formation, wound infection, bleeding, stomal necrosis, stomal retraction, mucocutaneous separation, and peristomal skin breakdown [3].

Late complications that develop after three months include stomal stenosis, peristomal skin breakdown, stomal prolapse, parastomal herniation, fistula formation, and negative psychological effects [3, 4]. Parastomal hernia is one of the common late complications affecting about 6.2% of patients with loop ileostomies. It is a type of incisional hernia resulting in the protrusion of abdominal content through the created defect. It presents with an abdominal swelling around the site of the hernia with or without pain, or as an acute strangulated or incarcerated hernia. [5].

Temporary stomas have negative effects on the quality of life (QoL). This is attributed to the fact that affected patients do not adapt well to a life with a stoma [6]. Some studies showed that patients who underwent low anterior resection with ileostomy had significant reductions in physical activity and role functioning [7]. Furthermore, it has negative effects on the body image. A pilot study showed that loop ileostomy patients had many concerns including feeling embarrassed about body image, feeling sexually unattractive, and limited choice of clothes, among other concerns. However, anxiety about pouch filling and loosening was among the biggest concerns. All these concerns were accredited to the lack of knowledge, lack of self-confidence about handling the stoma, fear of stoma getting injured, and the social taboo of having an “unusual thing on the body.”[8].

The reversal of loop ileostomies has an overall morbidity and mortality estimated at 17.3% and 0.4%, respectively [9]. To better understand how these complications may arise, it is beneficial to understand the steps involved in the reversal of a loop ileostomy.

Loop ileostomy reversal is as follows [7]:

- Prior to reversal of a diverting loop ileostomy, a water-soluble contrast enema is performed to demonstrate absence of any anastomotic leaks or other abnormalities in the downstream anastomosis.
- In the majority of cases, the reversal of the ileostomy is done through a local circumferential incision, encompassing 1-2 mm of skin surrounding the diverting loop ileostomy, at the level of the stoma itself, whereas a laparotomy is reserved only in the most extreme cases of tenacious adhesions.
- Then, each of the proximal and distal limbs is dismembered off the surrounding tissues all the way down into the peritoneal cavity.
- In order to avoid injury to the intestine, the rim of excised skin may be grasped with clamps to provide traction.
- The tip of the proximal limb is reinverted after a complete bowel mobilization, and the lumen of each of the limbs is injected under pressure with diluted Betadine solution or similarly coloured liquids mixed with air to check for occult leaks from inadvertent enterotomies.
- Then, the edges of the stoma are chipped to excise any residual skin and subcutaneous fat.
- The ileostomy is then closed, either by suturing the resulting in antimesenteric defect in one or two layers and by performing a side-to-side stapled enteroenteric anastomosis, among other techniques. The technique of ileostomy closure therefore mainly depends on the individual surgeon's preference.
- Following fascial closure, one technique is to partially reapproximate the skin using a subcuticular purse-string suture, which leaves a portion of subcutaneous tissue exposed to heal by secondary intention [7].

The morbidities associated with loop ileostomy reversal include small bowel obstruction (7.2%), anastomotic leak at the stoma closure site (1.4%), intraoperative bowel perforation and peritonitis (1.2%), and postoperative enterocutaneous fistula (1.3%). Other common morbidities include wound infection (5%) and incisional hernia in the stoma site (1.8%) [10]. These complications increase medical costs, prolong hospitalization time, and increase the need for outpatient care as well as the risk of late complications such as incisional hernia. Significant risk factors for complications after ileostomy reversal are male gender, surgical site infection, longer time from creation to closure, operation for diverticular disease with significant peritoneal contamination, age, type of ileostomy, the general condition of the patient, and small bowel resection [10].

Fortunately, some studies suggested that earlier closure is not only feasible but might reduce stoma-related morbidity, improve quality of life, and still effectively protect the distal anastomosis [11–14]. An improvement in the overall QoL is possible after ileostomy reversal, as Camilleri-Brennan and Steele illustrated. Their research showed that the reversal of the defunctioning ileostomy resulted in significant improvements in global QoL, physical function, social function, and role‐physical and energy‐vitality scores [15]. Gooszen et al. also demonstrated that there is a relation between the number of stoma care problems and the degree of social restriction, which significantly impacts the QoL [16].

Unfortunately, there are no established guidelines to indicate the optimal timing to reverse a loop ileostomy [17, 18]. Loop ileostomies reversal is generally recommended within 8–12 weeks after the primary surgery, in order to obtain adequate healing while avoiding an extended presence of a loop ileostomy with subsequent burden for the patient and the risk of developing stoma-related complications [17]. Nevertheless, because studies in population-based setting are rarely described in the surgical literature, it is uncertain how often loop ileostomies are reversed after 8–12 weeks in routine clinical practice [17].

In practice, temporary stomas may not be closed for months or even years. It may even become permanent. Hence, preoperative counselling and planning is just as important as it is for a permanent stoma [7]. The National Bowel Cancer Audit Project (NBOCAAP) 2013 report found that almost 30% of temporary ileostomies following anterior resection are still not closed after 18 months with the median time of closure being 7 months [17]. Other studies reported a risk of nonclosure between 3 and 25% [18]. Delayed reversal has various reasons including postoperative adjuvant chemotherapy, nonsurgical complications, symptomatic anastomotic leakage, and small bowel obstruction as well as administrative obstacles. This is explained by the fact that reversal operation is considered an elective low-priority operation that has to compete with more complex and urgent operations [17, 18].

Thus, the aim of this article is to review the relevant literature attempting to determine whether early closure of loop ileostomy is more beneficial compared to late closure (defined as closure >12 weeks after stoma creation) in terms of morbidity, mortality, and quality of life.

## 2. Materials and Methods

### 2.1. Study Selection

The relevant literature was selected through a multistep process. PubMed, Cochrane library, Google Scholar, and EBSCOhost search engines were searched using the terms “loop ileostomy,” “early closure,” and “late closure” and other possible phrasings including “delayed closure,” “early reversal,” and “delayed reversal” on their own or as a combination. The search yielded 2790 studies (161 results in Google scholar, 0 results in Cochrane library, and 2629 in EBSCOhost search engine). Then, the results were searched using our inclusion and exclusion criteria. Finally, the resultant articles included were collated and analysed in light of their results and quality.

Inclusion criteria were as follows:

- Adult subjects: 18 years old and older.
- English language.
- Full-text articles.
- Human subjects only.
- Studies that compared early versus late closure stoma-related outcomes, in terms of morbidity, mortality, and quality of life where available.
- Publications since 2008.

Exclusion criteria were as follows:

- Paediatric articles.
- Systematic reviews and meta-analysis.
- Studies that included only early or late closure stoma-related outcomes.
- Study protocols that did not include any results.
- Studies that did not meet with our selected definition of early and late closure timing.
- Studies that did not clearly link the rate of complications to the timing of closure.
- Abstract-only available.

There was no restriction placed on the type of research included in this review as long as it had a comparison of the outcomes of early and late closure of loop ileostomy (in accordance to our definition), in terms of morbidity, mortality, or QoL, which is our primary objective in this systemic review. The decision to review all types of literature with an obtainable comparison between the outcomes of early and delayed closure of loop ileostomy offered an opportunity to assess the quality of the different types of research on this topic. However, considering the nature of loop ileostomies, it was within our expectations that randomized controlled trials would be few.

Because loop ileostomies are done primarily to protect a downstream anastomosis, which is common among low rectal cancer patients, the decision has been made to include adults only as they are the primary affected population with low rectal cancer.

There is no available consensus regarding the definition of early and late closure of loop ileostomy. As such, it was decided to define late closure of loop ileostomy as any closure beyond three months of the stoma creation, in accordance with conventional literature [18]. This time interval would allow recovery after primary surgery, softening of intra-abdominal adhesion, and resolution of inflammation and edema of the stoma border [19]. The inclusion of only studies that included a comparison of early versus late closure of loop ileostomy outcomes was intended to provide more homogenous and comparable results.

### 2.2. Statistical Analysis

Microsoft excel was used for all data processing and representation.

## 3. Results

The search yielded 2790 studies (161 results in Google scholar, 0 results in Cochrane library, and 2629 in EBSCOhost search engine). The results were reduced to 147 after limiting them to full-text English publications since 2008 with human only subjects. Then, the results were searched using our inclusion and exclusion criteria (listed above), thus eliminating 143 nonrelevant studies. Finally, the four resultant articles included were collated and analysed in light of their results and quality.

A comparison between patient baseline demographics from these studies is shown in (Table 1), while some studies have demonstrated that early closure of loop ileostomy is feasible and beneficial [11, 20, 21]. Other studies did not report time to closure as a predictor of morbidity [22].

The postoperative complications overserved in each study were variable, and it is summarized as shown in Table 2.

Among the studies our search yielded, only two were multicentre randomized controlled studies including one that solely focused on comparing the outcomes of early and late closure of loop ileostomy with regard to the quality of life. The first randomized controlled study primarily compared the number of complications in early and late closure groups using computer-generated randomization by Danielsen et al. [14]. It included a total of 112 patients divided into early closure group (defined as closure 8–13 days after formation) and late closure group (defined as closure after >12 weeks). The study's primary endpoint was to compare both groups by examining the mean number of complications after total mesorectal excision (TME) for low rectal cancer. Secondary endpoints measured were the proportion of patients with at least one severe complication, in accordance with the Clavien–Dindo Classification, and the mean number of stoma-related complications after TME and up to 12 months, from the period of February 2011 to November 2015. The blinding of the intervention was not possible. The study noted that the patient baseline demographics, perioperative details of loop ileostomy closure, and its complications were comparable in both groups. It also noted that the mean number of complications after TME up to the 12 months of follow-up was significantly lower in the early closure group (mean number of complications was 1.24 in the early closure group compared with 2.88 in the late closure group) with a ratio for early to late closure of 0.42 (95% CI 0.32–0.57), *p* < 0.0001. As for more severe complications (Clavien–Dindo Grade IIIa or higher), there was no significant difference between the two groups (proportion of patients with at least one complication Clavien–Dindo Grade IIIa or higher; 0.22 for the early closure group and 0.29 for the late closure group, *p*=0.32). The mean number of stoma-related complications was significantly higher in the late closure group (early closure group 0.30 and late closure group 1.25) with a ratio for early versus late closure of 0.25 (95% CI 0.15–0.42), *p* < 0.0001.

The second multicentre randomized controlled study focused on comparing early closure (days 8–13 after stoma creation) with late closure (more than 12 weeks after stoma creation) of loop ileostomy with regard to QoL, by Park et al. [23]. The study evaluated health-related quality of life HRQOL at 3, 6, and 12 months after the index operation using the European Organization for Research and Treatment of Cancer (EORTC) questionnaires: QLQ-C30 and QLQ-CR29 and Short Form 36 (SF-36). These questionnaires aimed to measure physical, cognitive, emotional, and social functions. They also measured other items including financial difficulties, global health status, several symptoms, body image, sexual function, and interest, among others.

The study included 112 patients randomized to early or late closure group using computer-generated blocks of six, from February 2011 with the last follow-up in November 2015. The early closure group included 55 patients compared to 57 in the late closure group. The study noted the patient baseline demographics.

The response rates of the questionnaires were 82–95%, except for EORTC QLQ‐C30 at 12 months, to which only 54–55% of the patients responded due to an error in questionnaire distribution. The SF‐36 scores, overall, were similar between the two groups, with no differences in the physical component score or the mental component score. However, there were significant score differences in physical role at 3 months, bodily pain at 12 months, and mental health at 12 months. All dimensions in SF‐36 improved over time. The majority of patients in both groups scored values below mean levels in the reference population at 3 months, especially regarding physical role. At 12 months, 52–85% of the patients scored higher than the reference group, with physical functioning scoring the highest among the dimensions.

As for the EORTC QLQ‐C30 and QLQ‐CR29 questionnaires, the scores were comparable between early and late closure groups. Emotional functioning scores were lower in the early closure group at 3 and 6 months, but similar to the late closure group at 12 months. No statistically significant differences were seen in the dimensions of the QLQ‐CR29 questionnaire. In summary, there were no statistically significant differences in any questionnaire scores between the groups at 3, 6, or 12 months.

The only retrospective study in our data was carried in a single-centre, by Figueiredo et al. [24]. It included 259 rectal adenocarcinoma patients who underwent laparoscopic total or subtotal mesorectal excision (TME) with temporary ileostomy. These patients were divided into three groups, based on the time of stoma closure: group A included sixty-five patient who underwent stoma closure before day 61 after TME; group B included 115 patients who underwent stoma closure between day 61 and day 90; and group C included 79 patients who underwent closure after day 90. Both groups A and B patients fit into our definition of early closure of loop ileostomy but were dealt with separately in this article as grouping them was not possible without recalculating *p* values for the study. However, data needed for the process was not available. Patient baseline demographics were comparable in all groups and findings showed only 1 patient (1.5%) in group A and 2 patients (2%) from group B with anastomotic leakage compared to 44 patients (56%) in group C (*p* < 0.0001) post primary procedure. There were no significant differences between groups with regard to operative time, overall morbidity rate, and medical morbidity rates. However, one patient from group A and none from group B had anastomotic leakage at the site of stoma closure compared to four patients from group C (*p*=0.03). No patient from group A had stoma-related complications, whereas two cases of dehydration occurred in group B (2%) and two cases of acute renal failure occurred in group C (2.5%). Furthermore, one patient from group B had stoma prolapse and one patient from group C had peristomal evisceration, requiring surgery. At the time of stoma closure, 4 patients (6%) from group A and 14 patients (12%) from group B had a peristomal hernia compared with 21 patients (27%) in group C (*p*=0.001 group C versus group A and *p*=0.01 group C versus group B). Furthermore, group C had six patients with major morbidity (Clavien–Dindo III–IV) compared to zero patients in group B (*p*=0.004).

Median length of hospital stay after stoma closure in group C was 6 days [4–20] compared to 5 days [4–15] in group A (*p*=0.004). After a median follow-up of 17 [0–66] months, no significant difference between groups was observed for rehospitalization (3% in group A versus 3% in group B versus 8% in group C), bowel obstruction (0% versus 1% versus 1%), late anastomotic leakage (1% versus 3% versus 1%), or hernia at the stoma closure site (3% versus 11% versus 8%).

Finally, the last study in our data was a case matched study performed in a single-centre focusing on ileostomy closure before and after 3 months, by Li and Ozuner [25]. The study had a total of 358 patients and each group included 179 patients. As this was a case matched study, the characteristics of patient were similar in both groups; this includes the primary diagnosis, ASA, primary procedure, surgical technique used in primary and secondary procedure, and suspected complication from index procedure. The median time for ileostomy reversal in the early closure group was 64.5 ± 13.1 days, while closure in the late reversal group was 126.8 ± 44.2 days.

Postreversal outcome in both groups was similar and no statistical significance was demonstrated, including the rate of complications (see Table 1). For example, wound infection affected only 4 patients (2.2%) of the early group and 3 patients (1.7%) of the late group with a *p* value >0.99. This trend was observed in all complication rates including anastomotic leak, intra-abdominal abscesses, postoperative ileus, and small bowel obstruction.

## 4. Discussion

Loop ileostomies are created to protect distal anastomoses and reduce the impact of anastomotic complications. These are temporary stomas and their reversal is performed regularly. However, the length of time over which the stoma should persist is still debated. This study reviewed the literature to determine the effectiveness and safety of early closure of a defunctioning loop ileostomy compared to late closure, defined as closure of loop ileostomy after 12 weeks from the stoma creation, in terms of stoma-related morbidity, mortality, and quality of life.

The data in Danielsen et al. showed that early closure of a loop ileostomy is feasible, safe, and more effective in certain selected patients with rectal cancer compared to late closure. The study had a randomized design that included patients after ascertaining lack of asymptomatic anastomotic leakage. Due to the strict inclusion criteria of the study, not all patients were screened for participation. The study analysed the data of 112 patients out of 418 patients screened for participation. Thus, a selection bias may have occurred. Furthermore, the coding of complications during the study was done on twofolds. However, because it was not possible to blind the researchers who coded the actual complication, an observer bias may have also been undertaken. The baseline patient characteristics were comparable, thus excluding impact of age, gender, BMI, comorbidity, or neoadjuvant radiotherapy. Patients in the early closure group had fewer complications than patients in the late closure group, even during the follow-up of up to 12 months, with a ratio for late versus early of 0.42 (95% CI 0.32–0.57, *p* < 0.0001). Furthermore, there was no significant difference in the rate of severe complications (Clavien–Dindo Classification > IIIa) between the two groups (0.22 versus 0.29 for the early versus late closure groups, respectively; *p*=0.32). However, the severity of complications seemed higher in the late closure group.

Figueiredo et al. carried a retrospective single-centre study to include temporary stoma patients who had been radiologically screened within 5 weeks of stoma creation to exclude anastomotic leakage. The study included patients with adjuvant chemotherapy where the stoma closure took place 2-3 weeks after the last administration of chemotherapy. The study divided patients into three groups: A, B, and C, according to the time of closure. Both groups A and B patients fit into our definition of early closure of loop ileostomy. However, we were unable to group them together without recalculating the *p* values, which was not possible due to missing data. Due to the design of the study, it should be noted that a selection bias may have occurred. The patients baseline characteristics were comparable, thus excluding impact of age, gender, BMI, comorbidity, or neoadjuvant radiotherapy. The results of the study were in support of early closure of loop ileostomy. There was no significant difference in overall morbidity rate between the groups. However, significantly less patients from early closure groups (1 patient in group A and 0 patients in group B) had anastomotic leakage, at the site of stoma closure, compared with 4 patients from late closure group (*p*=0.03). Furthermore, late closure group had 6 patients with major morbidity (Clavien–Dindo III–IV) compared to none in early closure group (*p*=0.004). Even with ongoing adjuvant chemotherapy, stoma closure is safe, contrary to the conventional belief. This is demonstrated considering that the rate of surgical complications was higher in the late closure group despite a lower rate of ongoing chemotherapy (30% in group C versus 37% in group B). For example, it was observed that the parastomal hernia, at the time of stoma closure, was significantly more frequent in the late group (27%) than in the early closure groups (6 and 12% for groups A and B, respectively, *p*=0.001 versus group A, and *p*=0.01 versus group B). There were some issues regarding the lack of clear expanded definitions of the outcomes measured. For instance, the study included “late anastomotic leakage” as a measured outcome without clearly defining it.

Park et al. conducted a randomized study on the same population as Danielsen et al. discussed previously, as part of the EASY trial. However, this paper was focused on measuring the outcomes in terms of quality of life. The study clearly defined what clinically significant results were. However, the data showed no significant difference between early and late closure groups in terms of quality of life. All aspects of quality of life were similar between both groups and improved over time. The study failed to specify possible predictor factors affecting the quality of life such as the number of complications. It also neglected to account for the improvement of quality of life as time went by. These findings are contrary to other studies that clearly linked low anterior resection and stoma-related care problems to the quality of life, suggesting that early closure would be more beneficial [8, 17]. A significant limitation was that there were no baseline data for preoperative assessment due to the inclusion of patients only after rectal resection. Furthermore, this similarity between the groups may be explained by the fact that rectal cancer surgery requires a lengthy recovery, which would require a longer follow-up than 12 months for the significant differences to appear.

Wanglin Li et al. is a case matched study that included 358 patients. By definition, these studies have a retrospective aspect involved. In addition, due to the design, it is prone to be affected by selection and observer bias. It appears that the authors' logic from using such a design is to eliminate variables that could introduce bias between the study subjects. In other words, dividing the population into 2 equivalent groups in numbers and characteristics was intended to reduce the effect of variables on the final results. These included age, sex, pathology, comorbidities, primary surgery, and the same technique for stoma reversal. However, a strong factor that could influence the occurrence of complication is the surgeons technique which will always be variable.

It is worth noting that even though the authors were partially in favour of early reversal of loop ileostomies, their results are statistically inconclusive. In fact, the number of patients affected by each complication observed during the study was similar in the early and late closure groups. In addition, the study sample is small and the *p* values obtained were indicating statistical insignificance. Furthermore, the study did not take into consideration the social and psychological complications. This review is limited by the heterogenicity of the studies included. Another limitation was the small number of studies that met our criteria. This is due to our own selection of how to define late closure which was based on convention as there is no consensus on how to define late closure of loop ileostomy [18]. As a result, various studies with different definitions of late closure were overlooked in this review [12, 21, 26, 27]. Furthermore, all the studies in this review shared significant limitations due to the small sample size, selection bias, and the lack of clear definitions of the measured outcomes.

## 5. Conclusions

In conclusion, current available data were inclined toward early closure of loop ileostomies, especially in patients with uneventful postoperative course, but not sufficient to draw firm conclusions on the matter. As such, further studies are required to establish the optimal time of reversal for specific groups of patients, what factors may indicate a delay of the reversal, and to what extent.

## Figures and Tables

**Table 1:** Comparison between patient baseline demographics.

Reference	Number of patients			Gender (F : M)			Age (years)			ASA												Main diagnosis		Index operation		Adjuvant radio-chemotherapy		
										I			II			III			IV									
	EC		LC	EC		LC	EC		LC	EC		LC	EC		LC	EC		LC	EC		LC	EC	LC	EC	LC	EC		LC
Danielsen et al.	55		57	56 : 44		37 : 63	67		67	22%		33%	38%		23%	33%		35%	5%		2%	Rectal cancer		TME		29%		28%
Figueiredo et al.	65^a^	155^b^	79	45 : 55^a^	50 : 50^b^	34 : 66	60^a^	62^b^	62	28%^a^	29%^b^	20%	63%^a^	63%^b^	67%	9%^a^	8%^b^	13%	—^a^	—^b^	—	Rectal cancer		TME		75%^a^	58%^b^	59%
Wanglin et al.	179		179	88 : 91		88 : 91	45.7		45.6	56.4%^c^		56.4%^c^	56.4%^c^		56.4%^c^	44%^d^		44%^d^	44%^d^		44%^d^	Cancer (20.1%) IBD (69.8%) others (10.1%)		AC/LI (36.3%) CP/IPAA (31.3%) LAR/DLI (10.1%) TP/IPAA (6.7%) others (15.6%)	AC/LI (27.9%) CP/IPAA (30.7%) LAR/DLI (11.7%) TP/IPAA (6.7%) others (22.9%)	17%		17%
Park et al.	55		57	31 : 24		21 : 36	67		67	n/r		n/r	n/r		n/r	n/r		n/r	n/r		n/r	Rectal cancer		TME		69%		68%

^a^Closure before 60 days of stoma creation. ^b^Closure 61–90 days after stoma creation. ^c^Percentage of ASA I and II grouped together. ^d^Percentage of ASA III and IV grouped together. n/r: not reported; EC: early closure; LC: late closure; TME: total mesorectal excision; CP/IPAA: complete proctectomy with ileal-pouch anal anastomosis; LAR/DLI: low anterior resection/diverting loop ileostomy; TP/IPAA: total proctocolectomy with ileal-pouch anal anastomosis; IBD: inflammatory bowel disease.

**Table 2:** Comparison between the postoperative complications.

Complication	Reference								
	Danielsen et al.		Figueiredo et al.			Wanglin et al.		Park et al.	
	EC (*n* = 55)	LC (*n* = 57)	EC		LC (*n* = 79)	EC (*n* = 179)	LC (*n* = 179)	EC (*n* = 55)	LC (*n* = 57)
			<60 d (*n* = 65)	61–90d (*n* = 115)					
Infection	2	0	1	3	1	4	3	*n*/r	*n*/r
Parastomal hernia	*n*/r	*n*/r	3%^b^	11%^b^	8%^b^	*n*/r	*n*/r	*n*/r	*n*/r
Anastomotic stenosis	0	1	*n*/r	*n*/r	*n*/r	*n*/r	*n*/r	*n*/r	*n*/r
Reoperation	5^a^	4^a^	*n*/r	*n*/r	*n*/r	1	3	*n*/r	*n*/r
Small bowel obstruction	1	1	0^b^	1%^b^	1%^b^	6	3	*n*/r	*n*/r
Anastomotic leakage	0	1	1	0	4	1	1	*n*/r	*n*/r
Late anastomotic leakage	*n*/r	*n*/r	1%^b^	3%^b^	1%^b^	*n*/r	*n*/r	*n*/r	*n*/r
Ileus	*n*/r	*n*/r	1	4	4	22	24	*n*/r	*n*/r
Electrolyte disorder	*n*/r	*n*/r	4	3	1	*n*/r	*n*/r	*n*/r	*n*/r
Abscess	3^a^	2^a^	0	0	3	1	3	*n*/r	*n*/r
Postop bleeding	0	0	0	2	2	1	2	*n*/r	*n*/r
Nausea/vomiting	1	2	*n*/r	*n*/r	*n*/r	*n*/r	*n*/r	*n*/r	*n*/r
Pain	1	1	*n*/r	*n*/r	*n*/r	*n*/r	*n*/r	*n*/r	*n*/r
Allergy	0	0	*n*/r	*n*/r	*n*/r	*n*/r	*n*/r	*n*/r	*n*/r
Pancreatitis	0	0	*n*/r	*n*/r	*n*/r	*n*/r	*n*/r	*n*/r	*n*/r
Liver insufficiency	0	0	*n*/r	*n*/r	*n*/r	*n*/r	*n*/r	*n*/r	*n*/r
Cardiopulmonary	0	0	*n*/r	*n*/r	*n*/r	*n*/r	*n*/r	*n*/r	*n*/r
Urinary blockage	*n*/r	*n*/r	0	3	0	*n*/r	*n*/r	*n*/r	*n*/r
Urinary infection	*n*/r	*n*/r	0	1	1	*n*/r	*n*/r	*n*/r	*n*/r
Venous thromboembolism	*n*/r	*n*/r	0	0	1	*n*/r	*n*/r	*n*/r	*n*/r

*n*/r: not reported; EC: early closure; LC: late closure; ^a^within one-year follow-up; ^b^within 66 months of follow-up;
